# Hypoglycemic property of soy isoflavones from hypocotyl in Goto-Kakizaki diabetic rats

**DOI:** 10.3164/jcbn.17-68

**Published:** 2017-12-12

**Authors:** Ming Jin, Ming-hua Shen, Mei-hua Jin, Ai-hua Jin, Xue-zhe Yin, Ji-shu Quan

**Affiliations:** 1 Department of Biochemistry and Molecular Biology, Yanbian University Medical College, Yanji 133002, China; 2 Affiliated Hospital of Yanbian University, Yanji 133000, China

**Keywords:** soy isoflavone, hypocotyl, hypoglycemic, diabetes, GK rats

## Abstract

The present study was carried out to investigate the hypoglycemic effect of soy isoflavones from hypocotyl in GK diabetic rats. A single administration and long-term administration tests were conducted in GK diabetic rats to test the hypoglycemic effect of soy isoflavones. At the end of long-term administration trial, blood protein, cholesterol, triglyceride, glycosylated serum protein, C-reactive protein, insulin, aminotransferase, lipid peroxide, interleukin-6, tumor necrosis factor-α were estimated. Inhibition of soy isoflavones against α-amylase and α-glucosidase, as well as on glucose uptake into brush border membrane vesicles or Caco-2 cells were determined *in vitro*. In single administration experiment, soy isoflavones reduced postprandial blood glucose levels in GK rats. In long-term administration, hypoglycemic effect of soy isoflavones was first observed at week 12 and maintained till week 16. A significant reduction in fasting blood glucose, C-reactive protein, and lipid peroxide was noted at week 16. However, there was no significant treatment effect on blood insulin. Furthermore, soy isoflavone administration resulted in significant decreases in glycosylated serum protein, tumor necrosis factor-α, and interleukin-6. Other biochemical parameters, such as protein, cholesterol, triglyceride and aminotransferases were not modified, however. The results *in vitro* showed that soy isoflavones showed a potent inhibitory effect on intestinal α-glucosidase, but not on pancreatic α-amylase. Soy isoflavones also decreased glucose transport potency into brush border membrane vesicles or Caco-2 cells. It is concluded that soy isoflavones from hypocotyl, performs hypoglycemic function in GK rats with type 2 diabetes, maybe via suppression of carbohydrate digestion and glucose uptake in small intestine.

## Introduction

Diabetes mellitus is a metabolic disorder with hyperglycemia and insulin resistance, and has a considerable impact on life quality and life expectancy.^(1)^ It can be handled by exercise, proper diet and oral antidiabetic agents, and targeted glycemic levels will be achieved. However, these synthetic drugs are expensive and produce some undesirable side effects. Therefore, searching for relatively unexpensive, safe and effective antidiabetic agents is very important, especially for developing countries.^(2)^

Soybean (*Glycine max* L. Merr.) is known for containing various functional, non-nutrient phytochemicals, such as isoflavones, phytates and saponins.^(3)^ Among them, soy isoflavones are the most commonly noted components. Studies have shown that soy proteins and isoflavones moderate hyperglycemia in diabetic rats,^(4,5)^suggesting a protective effect of soybeans on diabetes mellitus. Report from Ali *et al.*^(6)^ also revealed that administration with soy isoflavones reduced blood glucose, while blood triglycerides unchanged in diabetic rats. Although these scientific data support the beneficial effect of soy and its isoflavone components on blood glucose control, the mechanism underlying their preventive effects on diabetes mellitus is still largely unknown.

In recent years, polyphenol-rich functional foods and the bioactive compounds have been developed to be potential preventive agents for diabetes.^(7)^ It is speculated that the diabetic progression could be blocked via the suppression of carbohydrate digestion and absorption in the gastrointestinal tract.^(7,8)^ So, one of the novel oral policies to alleviate hyperglycemia is the inhibition of α-amylase and α-glucosidase, as well as glucose absorption in the small intestine.^(8)^ In the small intestine, carbohydrates are degraded into polysaccharides and disaccharides by α-amylase, then further into monosaccharides by α-glucosidase, and finally, absorbed into small intestinal epithelial cells.^(8)^ Our study previously revealed that soy isoflavones are the potential inhibitors of yeast α-glucosidase,^(9)^ and Rasouli *et al.*^(7)^ also speculated that daidzein, one of the soy isoflavones, could inhibit the α-glucosidase using molecular docking. To further investigate the preventive role of soy isoflavones from hypocotyl (SIF) on postprandial hyperglycemia, we determined the effect of single and long-term administration of SIF on blood glucose control in Goto-Kakizaki (GK) diabetic rats.

The GK rats, non-obese diabetic animals with low insulin secretion, are the relevant animal model suitable for studying the human type 2 diabetes.^(2,10)^ Since intensive glycemic control is the major goal in treatment of diabetes and essential for reducing the risk of diabetic complications,^(11)^ it is worth studying the effect of soy isoflavones on glycemic control in GK rats with type 2 diabetes. The purpose of the present study was to assess the effect of chronic feeding of SIF on glycemic control in GK diabetic rats and evaluate its possible use as a hypoglycemic agent. We also focused on the hypoglycemic mechanism of SIF *in vitro*, including the inhibitory potentials on the carbohydrate digestive enzyme activities and glucose uptake in the small intestine.

## Materials and Methods

### Chemicals

Test kits for cholesterol, triglyceride and glucose were obtained from Wako Pure Chemical Industries (Osaka, Japan). Lipid peroxide (LPO) test kit was purchased from Kyowa Medex Company (Tokyo, Japan). ELISA kit for insulin were from Wako Pure Chemical Industries; and ELISA kits for glycosylated serum protein (GSP), C-reactive protein (CRP), interleukin (IL)-6, and tumor necrosis factor (TNF)-α were from BD Bioscience (San Jose, CA). Acarbose (ACR) was obtained from Wako Pure Chemical Industries; daidzein, daidzin, malonyldaidzin, glycitein, glycitin, malonylglycitin and malonylgenistin were purchased from Fujicco Company Ltd. (Kobe, Japan); genistein and genistin were obtained from Extrasynthese (Genay, France).

### Preparation and characterization of test extract

 Soybean seeds were obtained from the National Institute of Agrobiological Resources (Ibaraki, Japan); Soybean hypocotyl was shaken with 50% methanol overnight and the solution was filtered, and spray-dried. The yield (w/w) of the hypocotyl extract was 14.9% for the soybean seeds in terms of dry weight. The hypocotyl extract was then dissolved in 10% methanol and applied to ODS column (YMC, ODS-A60-S150, 5 cm × 74 cm).^(12)^ The column was eluted step-wisely with 10%, 30% and 80% methanol. Each fraction was collected, evaporated to dryness under vacuum and finally lyophilized.^(12)^

**Fig. 1:**
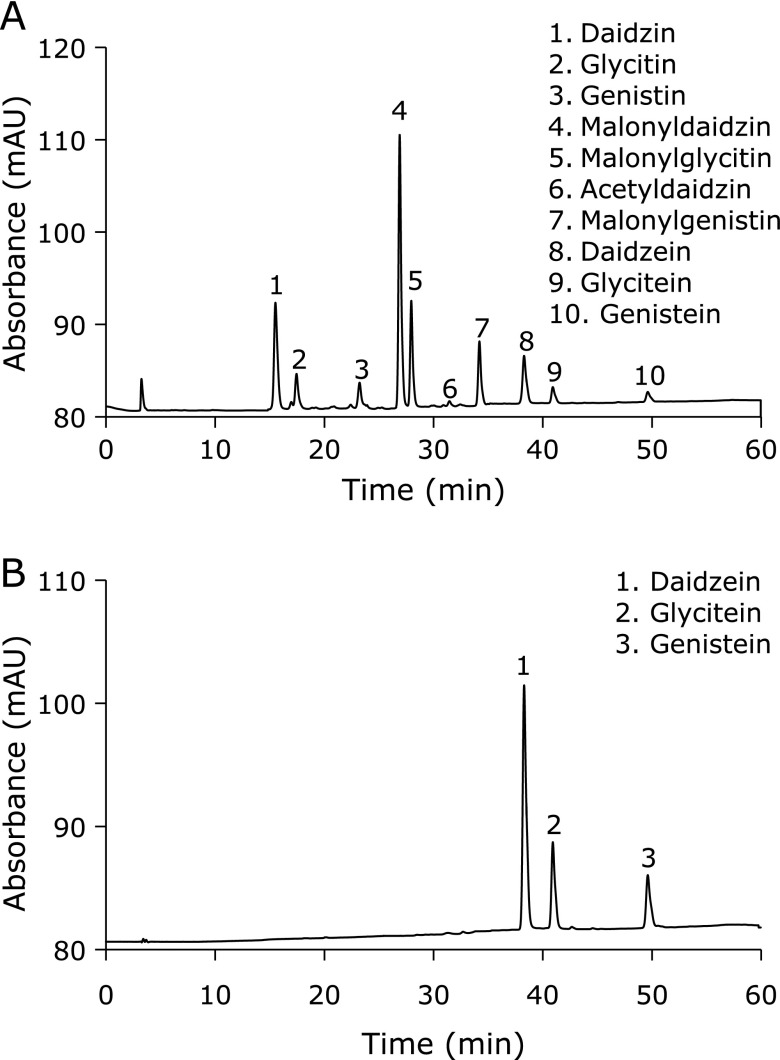
HPLC chromatograms of F_30_ (A) and SIF (B) showing isoflavone glycoside and aglycone peaks, respectively. HPLC was carried out using Waters 600E system. Column: YMC-packed ODS-AM-303 column (5 µm, 4.6 mm × 50 mm); UV-detection: 260 nm; mobile phase: a linear gradient of acetonitrile from 15% to 35% containing 1% trifluoroacetic acid.

**Fig. 2:**
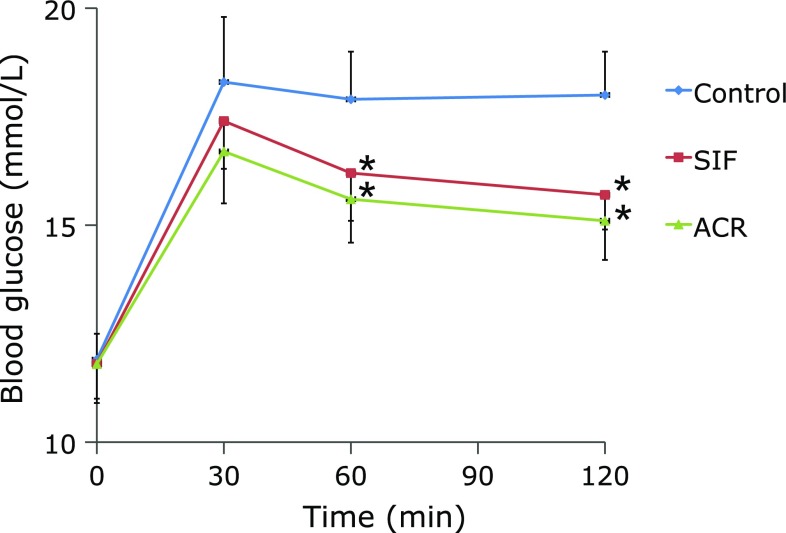
Effect of single administration of SIF on BG levels in maltose-loaded rats. Maltose (2 g/kg BW) and SIF (150 mg/kg BW) or ACR (3 mg/kg BW) were orally administered to the rats. The results are expressed as means ± SE of ten rats. *p<0.05, compared with control group.

To guarantee reproducibility of pharmacological experiments, isoflavone contents of fractions and SIF were analyzed with TLC and HPLC before further application.^(12)^ TLC was performed on a silica gel, and spots were visualized first by fluorescence scanning at 254 nm, then by sulfuric acid spraying and heating. HPLC was carried out using Waters 600E system as before.^(12)^ The results revealed that 10% methanol fraction consisted of soybean oligosaccharides; 30% methanol fraction (F_30_) consisted of malonylated soy isoflavone glycosides; 80% methanol fraction mostly consisted of soyasaponins and a small amount of isoflavones (non-malonylated isoflavone glycosides and isoflavone aglycones). Therefore, the mixture of F_30_ was used as precursor of SIF in this experiment. The yield (w/w) of F_30_ was 8.4% in terms of dry weight. The composition was as follows (%): malonyldaidzin 49.8, malonylglycitin 19.7, malonylgenistin 13.2, daidzin 2.5, glycitin 0.8, genistin 4.2, acetyldaidzin 1.1, acetylglycitin 0.5 (Fig. 1A). Finally, the above-mentioned F_30_ was hydrolyzed with 6 M hydrochloric acid solution for 4 h. The hydrolysate was extracted with ethyl acetate, distilled and freeze dried to obtain SIF powder. The composition of SIF was as follows (%): daidzein 61.7, glycitein 21.5, genistein 16.2 (Fig. 2B).

### Single administration test in normal Wistar and GK diabetic rats

Twenty-week-old male GK type 2 diabetic rats were randomly assigned to three groups: the control, ACR (positive control) and SIF treatment groups, each consisting of 10 animals. After overnight fasting, the control group received maltose solution at dose of 2 g/kg body weight (BW), whereas the maltose solution (2 g/kg BW) containing SIF (150 mg/kg BW) or ACR (3 mg/kg BW) was administered to SIF or ACR treatment groups, respectively. The dose of SIF treatment were selected based on our previous report,^(12)^ as well as its efficacy in obese Zucker rats.^(5)^ This dose of SIF is approximately equivalent to 3.5 g/kg BW of hypocotyl extract, and equal to 20 g/kg BW of soybean hypocotyl, in terms of dry weight.

Blood glucose (BG) was determined at 0, 30, 60 and 120 min after maltose load with a blood glucose test meter (Bayer, Leverkusen, Germany). During the oral maltose tolerance test, the glucose responses were calculated as the corresponding incremental integrated glucose values. Incremental areas under the curve (AUC) of changes in blood glucose were calculated by the following formula:^(2)^

AUC = Σ{[(C_i_ − C_0_) + (C_i+1_ − C_0_)] × (t_i+1_ − t_i_)}/2

### Long-term administration test in GK diabetic rats

 Six-week-old male GK type 2 diabetic rats were randomly assigned to the control, ACR (positive control) and SIF treatment groups, each consisting of 10 animals. The control group was fed on a standard laboratory chow, while SIF and ACR treatment groups were fed diets containing SIF (150 mg/kg BW) or ACR (3 mg/kg BW) for a period of 16 weeks. The composition of the standard diet was as follows (%). Corn starch 30.0, sucrose 25.0, casein 25.0, corn oil 10.0, cellulose powder 5.0, mineral mixture 3.5, vitamin mixture 1.0, methionine 0.3 and choline bitartrate 0.2.^(12)^ The mixtures of minerals and vitamins were according to AIN-93G MX and AIN-93G VX.^(13)^ SIF was added to the diet at the expense of starch. Rats were allowed free access to water and food, and water and food intake were monitered every day. Experimental animals were kept at 24 ± 1°C, under a natural light-dark regimen. Experimentations were performed in strict accordance with recommendations of Institutional Animal Ethics Committee of Yanbian University Medical College.

Blood samples were collected and tested for fasting BG at 4-week intervals. Fasting BG was measured with a blood glucose test meter (Bayer). An oral maltose tolerance test was carried out at week 12 and 16. On test days, animals were fasted overnight, and then received maltose solution at dose of 2 g/kg BW. Postpradial BG was determined at 0, 30, 60 and 120 min after maltose load, and the incremental AUC of BG levels were calculated as before.^(2)^

At week 16, rats were anesthetized, sacrificed, and then blood samples were withdrawn. Blood insulin, GSP, CRP, IL-6, TNF-α and LPO were assayed in accordance with the manufacturer’s instructions. Blood lipids and other parameters were detected with a biochemical autoanalyser (Hitachi 736-15, Tokyo, Japan) with respective commercial test kits.

### Carbohydrate digestive enzyme inhibitory assay *in vitro*

The small intestine of GK rat was dissected and washed out, the mucosa was scraped off and homogenized, and then the supernatant was used as a crude enzyme after centrifugation.^(8)^ The total α-glucosidase inhibitory assay was done by the method described by Watanabe *et al.*,^(14)^ and the total activity was measured by monitoring *р*-nitrophenyl released from *р*-nitrophenyl-glucoside at 400 nm. Activities of sucrase or maltase were determined by monitoring glucose liberated from sucrose or maltose at 505 nm with a Glucose B Test kit (Wako).^(8)^ Porcine pancreatic α-amylase suspension (Sigma, type 1-A, St. Louis, MO) was diluted and the activity was determined by monitoring starch at 700 nm by iodine method.^(8)^

### Inhibition of glucose uptake into intestinal brush-border membrane

Brush-border membrane vesicles (BBMVs) were prepared by the MgCl_2_ precipitation method from rabbit small intestine,^(15)^ and uptake of d-glucose was measured by a rapid filtration technique.^(16)^ The radioactivity was counted in a scintillation counter (LKB Wallac 1209 Rackbeta). Incubation was terminated at a time corresponding to peak glucose uptake. Time for peak glucose uptake varies among BBMV batches from 10 to 20 s.

### Inhibition of glucose uptake in intestinal epithelial cells

Caco-2 cells were obtained from Keygen Biotechnology (Nanjing, China). Cells were grown routinely in DMEM (Biological Industries, Kibbutz Beit-Haemek, Israel) containing 10% fetal bovine serum (Gemini Bio-Products, West Sacramento, CA), at 37°C in 5% CO_2_. Cells were grown to 80% confluence, and seeded at the density of 2 × 10^5^ cells/well on 12-well cell culture plates. d-glucose uptake was measured according to the previous report.^(8)^ The radioactivity in each monolayer was measured with a liquid scintillation counter (LKB Wallac 1209 Rackbeta, Turku, Finland).

### Statistical analysis

Results were presented as mean ± SE. For the single and long administration experiment, data were analyzed by two-way ANOVA followed by Dunnett’s post-hoc test using SPSS 20.0 software (SPSS Inc., Chicago, IL). In the long-term experiment, statistical significances of biochemical parameters were determined by one-way ANOVA followed by Tukey’s post-hoc test. Differences were considered as being statistically significant at *p*<0.05.

## Results

### Effects of single administration of SIF in GK diabetic rats

Figure 2 and Table 1 represent the results of the single administration test in GK diabetic rats. The BG levels peaked at 30 min after maltose load, and the postprandial BG levels following SIF or ACR treatments were reduced at 60 and 120 min after oral maltose load as compared with the control group (Fig. 2, *p*<0.05). These results suggest that SIF and ACR have the maltose intolerance ameliorating effects in GK diabetic rats. For GK diabetic rats, the total AUC was significantly lower in ACR group than the control group (Table 1, *p*<0.05). The total AUC of SIF group was tended to decrease, though the difference was not statistically significant as compared with the control group (Table 1, *p* = 0.09). No statistically significant differences of BG and total AUC were observed between SIF and ACR groups (Fig. 2, Table 1, *p*>0.05).

### Effects of long-term administration of SIF in GK diabetic rats

#### Assessment of BW, food and water intakes of the animals

**Fig. 3:**
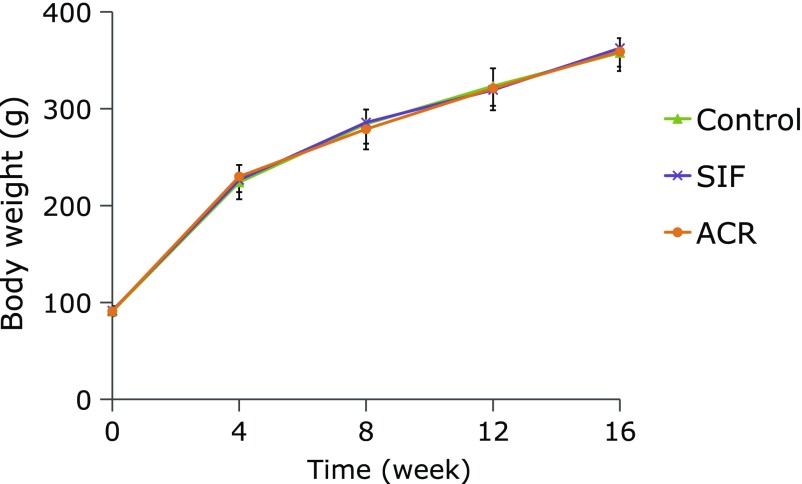
Effect of SIF on BWs of GK rats in long-term administration test. SIF (150 mg/kg BW) or ACR (3 mg/kg BW) were orally administered to the rats for 16 weeks. The results are expressed as means ± SE of ten rats.

**Fig. 4:**
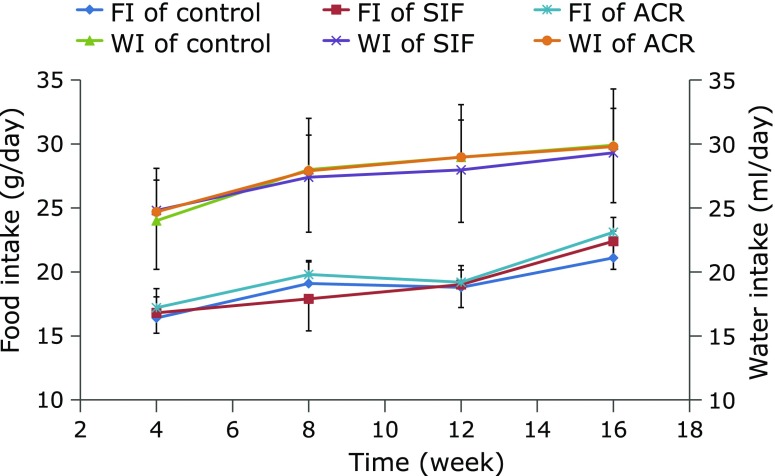
Effect of SIF on food intake (FI) and water intake (WI) of GK rats in long-term administration test. SIF (150 mg/kg BW) or ACR (3 mg/kg BW) were orally administered to the rats for a period of 16 weeks. The results are expressed as means ± SE of ten rats.

Throughout the experimental period, BWs of GK rats continued to increase in all groups, but no significant difference was observed among the rats from the control, SIF and ACR treatment groups (Fig. 3). Neither the water intake (WI) nor food intake (FI) was significantly different among the three groups during the experiment (Fig. 4).

#### Assessment of fasting BG and oral maltose tolerance

**Fig. 5:**
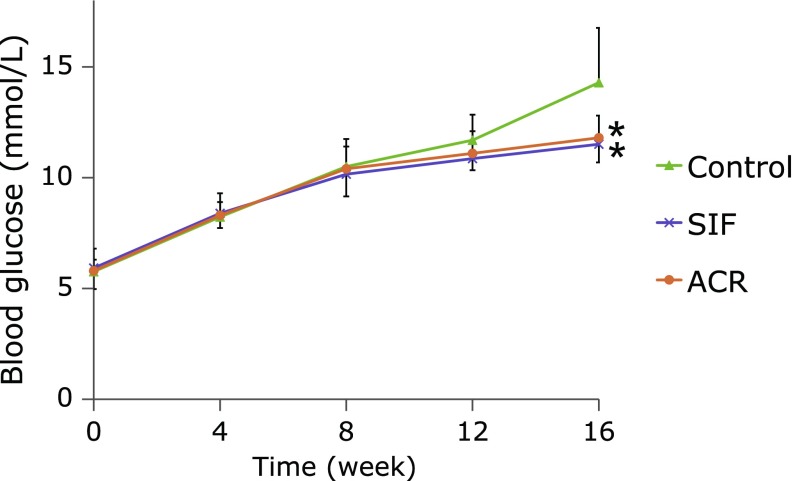
Effect of SIF on fasting BG levels of GK rats in long-term administration test. SIF (150 mg/kg BW) or ACR (3 mg/kg BW) were orally administered to the rats for 16 weeks. Blood samples were tested for fasting BG at 4-week intervals. The results are expressed as means ± SE of ten rats. *p<0.05, compared with control group.

 Figure 5 shows the effect of SIF on fasting BG in GK rats. The fasting BGs of all groups showed the tendency to elevate from week 0 to 16. At week 12, statistically insignificant reductions in fasting BGs were observed in SIF and ACR groups compared with the control group (*p*>0.05). However, chronic administration with SIF or ACR for a period of 16 weeks produced a significant reduction in fasting BG levels compared with the control GK rats (Fig. 5, *p*<0.05).

**Fig. 6:**
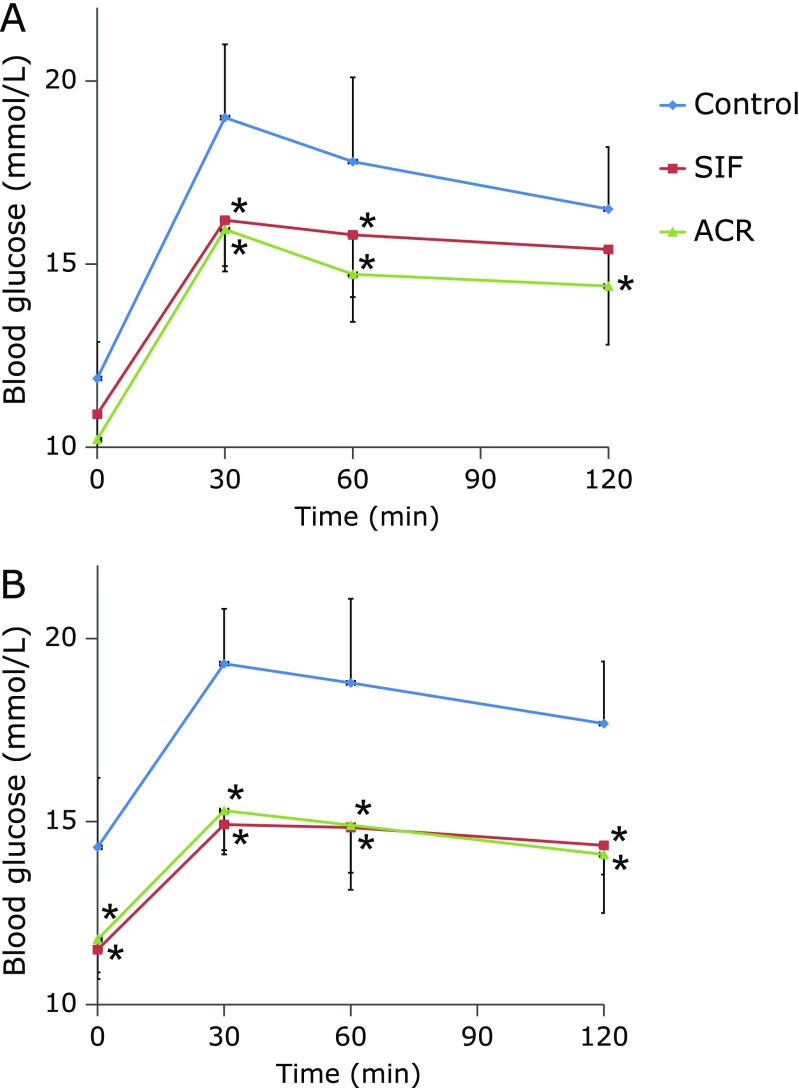
Effect of long-term administration of SIF on BG levels in maltose-loaded GK rats at week 12 (A) and 16 (B). SIF (150 mg/kg BW) or ACR (3 mg/kg BW) were orally administered to the rats for 16 weeks. At week 12 (A) and 16 (B), animals were fasted and an oral maltose tolerance test was carried out. The results are expressed as means ± SE of ten rats. * p<0.05, compared with control group.

In parallel with the amelioration of fasting BG levels, there were statistically significant reductions in postprandial BG levels in rats after long-term administration of SIF or ACR. Maltose intolerance ameliorating effects of SIF and ACR were apparently observed at week 12 and 16. As shown in Fig. 6, GK rats of the control group showed basal hyperglycemia and the hyperglycemia was aggravated by oral maltose challenge and failed to fall back to fasting BG level after 120 min, indicating impaired maltose tolerance. At week 12, the postprandial BG levels of the SIF and ACR treatment groups decreased significantly at 30 and 60 min (Fig. 6A, *p*<0.05), and total AUCs were tended to decrease, though the differences between the control and SIF or ACR treatment groups were not statistically significant, respectively (Table 2, *p*>0.05). At week 16, the maltose intolerance ameliorating effects of SIF and ACR were more clearly observed. Postprandial BG levels of SIF and ACR treatment groups were significantly lower than those of the control group at all time points checked (Fig. 6B, *p*<0.05), but there were no statistically significant differences of total AUCs among the three groups of diabetic rats (Table 2, *p*>0.05).

#### Assessment of insulin, GSP, LPO, CRP, TNF-α, IL-6 and other biochemical parameters

In this experiment, GSP level was also studied as an index of glycemic control in GK rats. At the end of the experiment, a small but significant reduction in GSP level was observed in GK rats administered with SIF or ACR as compared with the control GK rats (Table 3). However, no significant treatment effect was found for blood insulin of GK rats. In addition to a decrease in GSP level, the chronic SIF administration also induced a substantial modification in oxidative and inflammatory damages to the plasma. Compared with the control group, SIF or ACR treatment significantly decreased blood LPO, CRP, TNF-α and IL-6 levels (Table 3, *p*<0.05). The levels of blood total protein, triglyceride, cholesterol, HDL-cholesterol and other biochemical parameters, including AST, ALT, were not modified with SIF or ACR treatment, however (Table 3).

### Effect of SIF on carbohydrate digestive enzymes and glucose uptake *in vitro*

Inhibitory potentials of SIF against α-glucosidase and α-amylase were summarized in Table 4. SIF exhibited inhibitory activities on total α-glucosidase, including maltase and sucrase from rat small intestine. The inhibitory effects of isoflavone aglycones, the major components of SIF, and ACR were also shown in Table 4. However, the inhibitory activities of SIF on pig pancreatic α-amylase was so weak, with the inhibitory activities of 10 to 20% at the concentration of 1 g/L, that the half inhibitory concentration (IC_50_) values were not able to be detected in this experiment (Data were not shown).

**Fig. 7:**
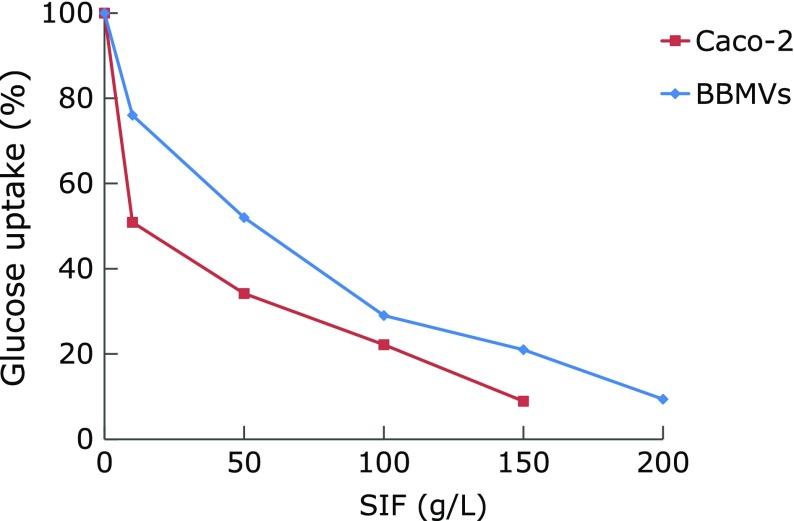
Effect of SIF on glucose uptake using BBMVs or Caco-2 cells *in vitro*. The results represent the means of six experiments.

In addition, SIF suppressed concentration-dependently glucose uptake into BBMVs or Caco-2 cells over the range of 5 g/L to 200 g/L, with the IC_50_ values of 49 and 12 g/L, respectively (Fig. 7). IC_50_ values of phlorizin, a glucose uptake inhibitor, were 1.6 and 0.48 g/L in these *in vitro* systems.

## Discussion

Recent studies have focused on soy phytochemicals, and isoflavones are the most commonly noted bioactive components among them.^(3)^ Soy isoflavones are mostly concentrated in soybean hypocotyls. They occur as glucose-conjugated forms and are hydrolyzed to absorbable aglycones once ingested.^(17)^ The bioavailability of aglycones and glucosides of soy isoflavones is controversial.^(18,19)^ However, it is commonly assumed that isoflavone aglycones are absorbed more quickly and in greater amounts than their glucoside forms.^(19)^ Our previous study indicated that soy isoflavone aglycones had an inhibitory activity against α-glucosidase,^(9)^ and that provides a strong rationale for further animal studies and encourages us to investigate the hypoglycemic effect of SIF in GK rats.

In the single administration experiment, we studied whether SIF would suppress the postprandial BG levels after oral maltose load. The results showed that the administration of SIF significantly suppressed the postprandial BG levels at 120 min after maltose load in GK diabetic rats, whereas only the AUC_60–__120__ __min_ of diabetic rats exhibited a significant reduction than the control group. The results of the single administration experiment confirmed the preventive role of SIF on hyperglycemia in postprandial state.

Since a single administration of SIF had a beneficial effect on blood glucose control in diabetes, we further investigated the long-term administration experiment in GK diabetic rats. In this study, chronic administration of SIF induced significant reduction in fasting BG levels and amelioration in oral maltose tolerance in GK diabetic rats. After 12 weeks of treatment with SIF, a maltose intolerance ameliorating effect of SIF was observed, though fasting BG lowering effect was not observed. Significant difference in fasting BG levels between the two groups was noted at week 16 after SIF feeding.

The animal dosage of SIF in this study could be converted to human equivalent dose of 24 mg/kg BW based on body surface area,^(20)^ and this amount is far in excess of usual dietary intakes of 20~30 mg/day isoflavone aglycones among Japanese or Korean populations.^(21,22)^ However, the advantage of ingesting high quantities of soy isoflavones in humans is questionable, given a curvilinear relationship between bioavailability and intake.^(23)^ Considering the decreasing bioavailability with increasing isoflavone intake, the beneficial effect of SIF on fasting blood glucose control are more likely to be achieved by multiple intakes of soy isoflavones rather than from a single highly enriched product, on the basis of the pharmacokinetics.^(23)^ So, an increased clinical efficacy in dietary intervention studies would be expected from a modest intake of soy isoflavones consumed throughout the day, without the potential for long-term negative effects.

We also investigated the effects of SIF on chronic glycaemic status in GK rats. Fasting or postprandial BG reflects only an instant glucose level, while GSP provides a time-averaged glucose level over 1~2 weeks, and may be a valuable parameter for the evaluation of the adequacy of diabetic BG control.^(24)^ During the experimental period, the GSP of SIF-treated diabetic rats decreased significantly as compared to the control rats. This result indicates that chronic glycaemic status in GK diabetic rats were controlled by SIF treatment and this was consistent with the suppression of fasting and postprandial BG levels as described above.

However, this hypoglycemic effect of SIF was not associated with BW, food consumption and water intake, suggesting that SIF has an anti-hyperglycemic potential without affecting energy metabolism. No difference in blood insulin level was either observed between the two groups of GK rats. GK type 2 diabetic rats continue an experimental diabetes model of lack of insulin due to impaired glucose recognition by pancreatic β-cells.^(25)^ In this study, SIF did not stimulate insulin secretion of GK rats. These results suggest that SIF has the ability to lower BG without the help of endogenous insulin, and the suppression of the BG levels by SIF may be mediated through other pathways.

The major goal of diabetic treatment is to optimize glycemic control to prevent the diabetes-associated complications caused by hyperglycemia.^(11)^ Under hyerglycemic conditions, reactive oxygen species (ROS) are produced via glucose auto-oxidation and no-enzymatic protein glycation in tissues.^(26)^ Type 2 diabetes mellitus is also associated with innate immune activation, and exhibit increased TNF-α, IL-1β, IL-6 and CRP following IL-6 secretion.^(26)^ Therefore, phytochemicals for treatment of diabetes should possess both the anti-oxidative and anti-inflammatory effects.^(27)^ Beyond an improvement of glycemic control, the chronic SIF administration also induced a substantial modification in indexes of oxidative damage and inflammation to the blood. A significant decrease was apparent for blood LPO, CRP, TNF-α and IL-6 levels while there was no significant change of other blood biochemical parameters such as total protein, cholesterol, triglyceride, AST and ALT in SIF group compared with the control group. These results partly agree with the findings of Watanabe *et al.*,^(28)^ who have reported soybean hypocotyl tea had an antioxidant activity in humans.

A surge in BG is due to hydrolysis of dietary starch by digestive enzymes such as α-glucosidase and α-amylase, as well as glucose uptake by the intestinal tract.^(29,30)^ Polyphenol compounds have been proposed to be potential inhibitors of α-amylase and α-glucosidase, and inhibition of these enzymes can regulate carbohydrate metabolism.^(7)^ To investigate the hypoglycemic mechanism of SIF, we examined the inhibitory effects on carbohydrate digestive enzymes *in vitro*. Our data showed that SIF had inhibitory effects on α-glucosidase, including maltase and sucrase, which are present in the small intestinal mucosa, even though their inhibitory effects on these enzymes were weaker than those of acarbose. However, SIF displayed a much weaker inhibition potential towards pig pancreatic α-amylase. These results are in good agreement with the virtual screening studies of Rasouli *et al.*,^(7)^ who speculate that daidzein could inhibit the α-glucosidase but not α-amylase using molecular docking and virtual screening studies. Inhibition of these carbohydrate digestive enzymes can inhibit intestinal digestion of starch and suppress the BG levels in postprandial state, and could be used without the risk of hypoglycemia.^(30)^

However, inhibition of these carbohydrate digestive enzymes could not prevent glucose uptake in the digestive tract when foodstuffs contain glucose as sugar. So, it might be essential to block the intestinal glucose uptake to suppress the postprandial hyperglycemia. Glucose uptake in the small intestine is mediated by sodium-dependent glucose transporters (SGLTs), and any glucose in the lumen is rapidly taken up by SGLTs.^(8)^ To explore the possible influence of SIF on glucose absorption in intestinal tract, we examined glucose uptake by BBMVs or Caco-2 cells after treating with SIF. Phlorizin, a competitive inhibitor of SGLTs,^(8)^ was used as the positive control in this study. Phlorizin is used as a potential pharmaceutical treatment for type 2 diabetes mellitus, and also found to exist in black soybean, recently.^(31)^ We found that SIF suppressed concentration-dependently glucose uptake into BBMVs or Caco-2 cells, even though its inhibitory effect on glucose uptake was much weaker than that of phlorizin. Although these values are quite higher than the concentrations for digestive enzyme inhibition, they may yet be relevant *in vivo* since levels of these components in intestinal tract will be much higher than those in blood.^(32)^

In conclusion, SIF has a preventive effect against hyperglycemia in GK rats with type 2 diabetes mellitus, which may be related to suppression of carbohydrate digestion and glucose uptake in the small intestine. These findings demonstrate that soybean and soy isoflavones are potential candidates for medical food or pharmaceutical supplements in treatment of type 2 diabetes mellitus.

## Figures and Tables

**Table 1:** The incremental AUC of BG levels in single administration test

Time period (min)	Blood glucose AUC (mM·min)		
	Control	SIF	ACR
0–30	96.2 ± 16.5	84.7 ± 12.3	73.3 ± 14.3
30–60	185.6 ± 30.8	151.1 ± 22.4	129.2 ± 26.4*
60–120	362.5 ± 52.4	270.1 ± 40.3*	218.3 ± 35.2*
Total	644.3 ± 94.3	505.7 ± 71.3	420.8 ± 61.5*

Maltose (2 g/kg BW) and SIF (150 mg/kg BW) or ACR (3 mg/kg BW) were orally administered to the rats. The results are expressed as means ± SE of ten rats. *p<0.05, compared with control group.

**Table 2:** The incremental AUC of BG levels of GK rats in long-term administration test

Week	Time period (min)	Blood glucose AUC (mM·min)		
		Control	SIF	ACR
12	0–30	106.9 ± 17.8	79.5 ± 16.2	86.0 ± 11.3
	30–60	195.7 ± 32.2	153.4 ± 24.1	153.7 ± 19.5
	60–120	316.3 ± 56.1	281.7 ± 33.6	261.0 ± 35.4
	Total	618.8 ± 93.9	514.2 ± 57.4	500.7 ± 53.5
16	0–30	75.3 ± 23.6	51.3 ± 12.4	52.3 ± 13.3
	30–60	142.7 ± 36.2	101.5 ± 29.7	99.7 ± 28.4*
	60–120	236.3 ± 68.5	185.9 ± 41.3	163.3 ± 36.2
	Total	454.3 ± 82.7	338.8 ± 76.3	315.3 ± 61.5

SIF (150 mg/kg BW) or ACR (3 mg/kg BW) were orally administered to the rats for 16 weeks. At week 12 (A) and 16 (B), animals were fasted and received maltose (2 g/kg BW), and an oral maltose tolerance test was carried out. The results are expressed as means ± SE of ten rats. *p<0.05, compared with control group.

**Table 3:** Effect of SIF on blood biochemical parameters of GK rats in long-term administration test

Indexes	Control	SIF	ACR
Total protein (g/dl)	6.2 ± 0.3	6.1 ± 0.3	6.3 ± 0.2
Triglyceride (mM)	0.22 ± 0.03	0.27 ± 0.04	0.25 ± 0.03
Free fatty acid (mM)	0.73 ± 0.04	0.69 ± 0.06	0.62 ± 0.03
Cholesterol (mM)	2.7 ± 0.5	2.7 ± 0.6	2.9 ± 0.4
HDL-cholesterol (mM)	1.9 ± 0.3	1.9 ± 0.4	2.1 ± 0.4
LDL-C (mM)	0.81 ± 0.23	0.76 ± 0.14	0.86 ± 0.14
AST (U/L)	126 ± 19	134 ± 27	130 ± 26
ALT (U/L)	124 ± 25	128 ± 29	122 ± 23
Insulin (mU/L)	59.4 ± 18.3	64.4 ± 18.7	71.9 ± 20.1
GSP (mM)	1.54 ± 0.17	1.31 ± 0.19*	1.37 ± 0.16*
CRP (mg/L)	1.78 ± 0.23	1.20 ± 0.24*	1.39 ± 0.16*
TNF-α (µg/L)	81.5 ± 11.6	75.1 ± 8.6	70.3 ± 9.2*
IL-6 (ng/L)	383 ± 43.1	290 ± 25.4*	325 ± 28.5*
LPO (µM)	11.4 ± 2.0	9.7 ± 1.8*	8.1 ± 0.7*

SIF (150 mg/kg BW) or ACR (3 mg/kg BW) were orally administered to the rats for 16 weeks. At week 16, rats were anesthetized, sacrificed, and then blood samples were tested for the biochemical parameters. The results are expressed as means ± SE of ten rats. *p<0.05, compared with control group.

**Table 4:** IC_50_ values of SIF on rat α-glucosidase *in vitro*

Component	IC_50_ (g/L)		
	Total α-glucosidase (*K*_m_ = 0.7 mM)	Maltase (*K*_m_ = 1.7 mM)	Sucrase (*K*_m_ = 21.2 mM)
F_30_	>10	9.4	8.8
SIF	3.1	5.6	8.6
Daidzein	2.52	4.7	8.5
Genistein	2.39	3.9	9.4
Glycitein	8.8	8	>10
ACR	0.12	0.19	0.34

*K*_m_ values were obtained from Lineweaver-Burk plots of rat α-glucosidase. The results represent the means of four experiments.
